# Ultrasound-guided percutaneous tracheostomy: a feasible alternative for tetanus patients

**DOI:** 10.1186/cc13946

**Published:** 2014-06-26

**Authors:** Daniel Joelsons, Yeh-Li Ho, Marcelo Park

**Affiliations:** 1 Intensive Care Unit, Infectious and Parasitic Diseases Department, Hospital das Clinicas, University of São Paulo, Av. Dr. Enéas de Carvalho Aguiar, 255, 4° Andar, Infectologia, São Paulo 05403-900, Brazil; 2 Intensive Care Unit, Emergency Medicine Discipline, Hospital das Clínicas, University of São Paulo, Av. Dr. Enéas de Carvalho Aguiar, 255, 4° Andar, Infectologia, São Paulo 05403-900, Brazil

## 

Tetanus is a treatable and preventable disease that remains prevalent in developing countries. The mortality rate can reach up to 30 %. The early mortality is due to acute respiratory failure secondary to muscle spasms. Given that the tracheal tube stimulates muscle spasms, there is worldwide consensus to perform early tracheostomy on tetanus patients who need orotracheal intubation. Bronchoscopy-guided percutaneous tracheostomy is considered high-risk once it is likely to cause tracheal spasms [1-3].

The São Paulo government has recommended that all cases of tetanus in the São Paulo State be transferred to our ICU, which offers one of the best outcomes in tetanus treatments in Brazil. Usually, after orotracheal intubation, the tracheostomy is immediately performed by a thoracic surgeon; however, frequently, the procedure is delayed because of a shortage of surgeons.

Ultrasound-guided percutaneous tracheostomy (US-PCT) is a safe, easy, and fast method in several populations, including obese patients [2,4,5]. However, to our knowledge, there are no published data on US-PCT in patients with tetanus. We have just performed the first US-PCT on a patient with tetanus.

The US-PCT was performed after deep analgesia, sedation, and a neuromuscular blocker. Before the procedure, ultrasound was used to identify the structures, to verify whether there were any vessels in the area, and to locate the puncture site. The patient was ventilated under 100% fraction of inspired oxygen, and the endotracheal tube was retracted near the vocal cords under direct laryngoscope. An ultrasound-guided perpendicular puncture of the anterior trachea wall was made between the second and third tracheal cartilages. Once the needle passed through the anterior trachea wall, it was caudally angled to prevent any lesions of the posterior wall. The guide wire was inserted followed by a gentle dissection with a mosquito clamp until direct visualization of the pre-tracheal membrane. A second bolus of rocuronium was made, and only then was the dilator used in order to create the initial stoma followed by larger dilatation with the Griggs forceps and introduction of the tracheostomy tube. The whole procedure was accomplished in the ICU in 11 minutes and there were no complications.

The achieved result confirms the feasibility of this procedure in tetanus. We would like to emphasize some special care in US-PCT in this population - continuous intravenous diazepam and a second bolus of neuromuscular blocker before dilatation of the trachea.

### Abbreviations

US-PCT: ultrasound-guided percutaneous tracheostomy.

### Competing interests

The authors declare that they have no competing interests.
